# Supplementary material for: Revealing the History and Mystery of RNA-Seq
Source: Curr Issues Mol Biol. 2023 Feb 24;45(3):1860–74. doi: 10.3390/cimb45030120 (PMC10047236; doi:10.3390/cimb45030120)
Supplement: Supplementary file 1 [file cimb-45-00120-s001.zip › cimb-2206633-supplementary.pdf]

Supplementary Document (R-scripts)

Call the libraries

```
library(ggplot2)
library(ggrepel)
```

########## Volcano plot for Transcriptomics data ###########

```
de <- read.table("YourFile", header = TRUE, sep = "\t")
ggplot(YourFile, aes(x=log2_FC, y=-log(P_value)))+
  geom_point(aes(color="black", size=0.5, alpha= 1)) +
  labs(y=expression('-Log'[10]*' P-Value'), x=expression('Log'[2]*' fold change'))+
  scale_color_manual(values=c('black'))+ theme_bw()+
  theme(legend.position = "none",panel.grid.major = element_blank(), panel.grid.minor = element_blank(), panel.background = element_blank(),axis.line = element_line(colour = "black"), )
```

############ GSEA #################

```
library(tidyverse)
library(dplyr)
library(fgsea)


ranks <- tibble::deframe(YourFile)
head(ranks, 20)
```

# Load the pathways into a named list

```
pathways.hallmark <- gmtPathways("h.all.v7.5.1.symbols.gmt")
```

#pathways.hallmark

# Show the first few pathways, and within those, show only the first few genes.

```
pathways.hallmark %>%
  head() %>%
  lapply(head)
```

# run the fgsea algorithm with 1000 permutations:

```
fgseaRes <- fgsea(pathways=pathways.hallmark, stats=ranks, scoreType = "std")

fgseaResTidy <- fgseaRes %>%
  as_tibble() %>%
  arrange(desc(NES))
```

# Show in a nice table:

```
fgseaResTidy %>%
  dplyr::select(-leadingEdge, -ES) %>%
  arrange(padj) %>%
  DT::datatable()
```

#Plot the normalized enrichment scores. Color the bar indicating whether or not the pathway is significant:

```
ggplot(fgseaResTidy, aes(reorder(pathway, NES), NES)) +
  geom_col(aes(fill=padj<0.05)) +
  coord_flip() +
  labs(x="Pathway", y="Normalized Enrichment Score",
       title="Hallmark pathways NES from GSEA") +
  theme_minimal()

head(fgseaRes[order(padj), ])
```

###GSEA pathway enrichment plot ###

```
plotEnrichment(pathway =
               pathways.hallmark[["HALLMARK_MYC_TARGETS_V1"]],
             stats= ranks) + labs(title="HALLMARK_MYC_TARGETS_V1 ")+
  theme(plot.title = element_text(hjust = 0.5, face="bold"))
```

########## **MA plot** ######

```
library("DESeq2")
plotMA(YourFile)
```

heatmap

```
library("pheatmap")
setwd("YourDirectory")
de <- read.table("YourFile", header = TRUE, sep = "\t")
data_de <- melt(de)

ggplot(data_de, aes(variable,Gene)) +geom_tile(aes(fill = value))+ scale_fill_gradient(low = "red",
high = "green")
```

boxplot jitter

```
data_mod <- melt(YourSummary_AllEvents_rMATS ,
id.vars=colnames(YourSummary_AllEvents_rMATS),
              measure.vars=c("A3SS","A5SS","MXE","SE","RI"))

ggplot(data_mod, aes(x = variable, y = value))+
  geom_boxplot(outlier.shape = NA) +ylim(-1,1)+
  geom_jitter(alpha=0.4, aes(color=variable))+
  coord_flip()+
  theme_bw()
```
